# Clinical comparative effectiveness of acupuncture versus manual therapy treatment of lateral epicondylitis: feasibility randomized clinical trial

**DOI:** 10.1186/s40814-019-0490-x

**Published:** 2019-09-07

**Authors:** Katrine Bostrøm, Sverre Mæhlum, Milada Cvancarova Småstuen, Kjersti Storheim

**Affiliations:** 1 Norwegian Institute of Sports Medicine (NIMI), Sognsveien 75D, O805 Oslo, Norway; 2 Faculty of Medicine, Institute of Health and Society, University of Oslo, Oslo, Norway; 3 Faculty of Health Sciences, Oslo Metropolitan University, Oslo, Norway; 4 Research and Communication Unit for Musculoskeletal Health (FORMI), Oslo University Hospital, Oslo, Norway

**Keywords:** Tendinopathy, Eccentric exercise, Physical therapy

## Abstract

**Background:**

Lateral epicondylitis (LE) is a challenging condition for clinicians, and research has yet not proven the superiority of one specific treatment approach. However, manual therapy (elbow mobilization) in addition to eccentric exercise has been found to be superior to exercise alone. As well, acupuncture is effective in short-term pain relief when compared with sham treatment, but there is little knowledge on the comparative effectiveness of manual therapy and acupuncture treatment of LE in terms of pain relief. The primary objective of this pilot trial was to assess the feasibility (retention and adherence rates) of performing a randomized controlled trial (RCT) to explore the clinical effectiveness of acupuncture and manual therapy treatment of LE.

**Methods:**

This pilot trial took place in an outpatient interdisciplinary institute of sports medicine and rehabilitation in Oslo, Norway. Thirty-six adults with clinically diagnosed LE were randomly allocated into one of three groups: eccentric exercise alone, eccentric exercise plus acupuncture, or eccentric exercise plus manual therapy for a 12-week treatment period. Primary outcomes were patient retention and adherence rates. Secondary outcomes included patient-reported pain (NRS), level of disability (Quick-DASH), and participant’s satisfaction with treatment and global perceived effect.

**Results:**

Nine (69%) patients in the acupuncture group completed the 1-year follow-up, compared to eight (67%) in the manual therapy group and five (45%) in exercise alone. Our goal was to demonstrate a retention rate above 80% to avoid serious threats to validity, but the result was lower than expected. The majority of participants (64%) in both treatment groups received only three-treatment sessions; the reasons included non-attendance or recovery from pain. Secondary outcomes support the rationale for conduction of an RCT. There were no adverse advents related to study participation.

**Conclusions:**

Based on differences in pain relief between groups, patient retention, and adherence rates, an RCT seems to be feasible to assess treatment effectiveness more precisely. In a future definitive trial, greater dropout may be reduced by maintaining contact with the participants in the exercise alone group throughout the intervention, and objective assessments might be considered.

**Trial registration:**

ClinicalTrials.gov, NCT02321696

## Introduction

Work-related upper extremity disorders are a common problem in working populations in Western countries. They include a range of symptoms and afflictions related to the neck, shoulder, elbow, and hand [1]. Lateral epicondylitis (LE), or *tennis elbow*, is the most common chronic musculoskeletal pain condition affecting the elbow [2]. The annual incidence is 4 to 7 cases per 1000 patients in general practice [3] and is as high as 17% among workers in industries requiring highly repetitive hand motions [4, 5]. It is a painful condition, leading to loss of function in the affected limb, and can therefore have a major impact on patients’ professional and personal lives. LE persists for an average of 6 to 24 months [2]. It is further associated with significant sickness absence in 5% of affected working-aged adults [4, 5]. The cost is therefore high, both in terms of loss of productivity and health care utilization [1].

For some time, it was suggested that LE involved an inflammatory process, hence the name. Consistent absence of inflammatory cells has resulted in the consensus that the process is non-inflammatory in nature, and it has been redefined as degenerative [2, 6]. The main theory is that LE is caused by an incomplete repair of repetitive micro-trauma of the common extensor tendon tissue attached to the lateral epicondyle of the elbow, as in tendinopathy [6, 7]. Since LE often persists or recurs beyond the normal time for healing, it is recommended to speed up this healing process with physical treatments [8, 9]. An exercise program is the most common treatment in the management of LE, but the optimal exercise protocol is still unknown [3, 8, 9]. There is some evidence that eccentric exercise is superior to concentric exercise [10–12].

Over the past 10 years, the treatment of pain with acupuncture has gained wider acceptance among both clinicians and consumers of health care [13, 14]. Acupuncture is known to induce analgesia via several pain mechanisms [15]. There is some evidence suggesting that acupuncture treatment compared with sham acupuncture is effective in short-term pain relief (follow-up < 4 weeks) for patients with LE [16].

Research on physiotherapy treatment supports the suggestion that manual therapy techniques (Mulligan’s mobilization with movement) provide short-term pain relief for patients with LE, and combined with eccentric exercise, they are superior to a “wait and see” approach [17, 18].

At the time the protocol was planned, there were no published trials comparing acupuncture with manual therapy treatment of LE in terms of pain relief, and very few interventions have demonstrated the consistent effectiveness of any treatment. There appears to be a lack of evidence for the superiority of any specific intervention [7]. The effectiveness of the exercise program is low when applied as monotherapy [10]. Therefore, exercise for the treatment of LE is combined with other physiotherapy modalities like stretching, soft tissue mobilization, manual therapy, or acupuncture [9, 10].

The aim of the present study was to assess the feasibility of performing a randomized controlled trial (RCT) to explore the clinical effectiveness of acupuncture and manual therapy treatment of LE, both in addition to eccentric exercise and in comparison to a control group receiving eccentric exercise alone.

## Methods

### Trial design and setting

To prepare for a full-scale trial, a feasibility RCT was conducted in a private, interdisciplinary outpatient health care setting (NIMI) in Oslo, Norway. The design is a three-armed RCT [19]. The trial adheres to the principles of the Helsinki Decaration [20] and to the CONSORT guidelines for randomized pilot and feasibility trials [21]. The Regional Committees for Medical Research Ethics in South East Norway (Rek Sør-Øst B) (ref. no. 2014/1520) approved the project before the trial began. The trial was also reported to the Norwegian Centre for Research Data (NSD) and registered at ClinicalTrials.gov under the identifier NCT02321696 before commencing. All patients gave their written informed consent.

### Participants

Adults 18–67 years old referred to physiotherapists or medical doctors at NIMI with pain from the lateral part of the elbow were screened for eligibility. To be included, the patients had to report pain with an intensity of 4 or higher on a numeric rating scale (NRS; 0–10). Further inclusion criteria were pain on palpation, increased pain on resisted dorsiflexion of the wrist with the elbow extended and the fingers flexed, and resisted extension of the third finger [3]. To avoid light, self-limiting conditions being included, we pragmatically chose to exclude patients with symptom duration of less than 2 weeks. Other exclusion criteria were treatment with corticosteroid injection within the last 4 weeks, bilateral symptoms, radio-ulna or radio-humeral osteoarthritis, neck or shoulder problems, inflammatory rheumatic disease of the central or peripheral nervous system, or unwillingness to participate in the study.

### Baseline assessment

After informed consent was obtained, patients completed a standard questionnaire prior to randomization. The questionnaire included patient demographics, level of education, occupation, and previous cortisone injections and patient-reported outcomes. A physical therapist or a medical doctor at NIMI performed a clinical examination to assess eligibility.

### Randomization

We enrolled the patient in the study if all inclusion criteria and no exclusion criteria were met. Only then was the project leader contacted and asked to allocate the patient to one of three treatment groups: eccentric exercise alone, acupuncture in addition to eccentric exercise, or manual therapy in addition to eccentric exercise. The randomization was organized in blocks of six with a 1:1:1 ratio. Patients drew a sealed opaque envelope containing disclosure of group allocation from a collection of at least six envelopes. To prevent possible manipulation of group assignment, additional randomization envelopes were constantly added to avoid ending up with only one envelope left at the end of a block. For this reason, there was also an extra block that was added towards the end, which meant that the distribution was not 12 + 12 + 12.

### Interventions

During a 12-week treatment period, patients received one of three treatments: eccentric exercise alone, acupuncture in addition to eccentric exercise, or manual therapy in addition to eccentric exercise.

#### Eccentric exercise

We instructed all patients to follow an eccentric exercise program for LE in order to strengthen the extensor muscles and tendon [22, 23]. Strengthening exercises are a common treatment in the physical rehabilitation of tendon problems [9, 10]. To gain the maximum benefit from this exercise, the starting weight should be tailored individually; however, to simplify clinical application, the starting weight in this study was standardized. Participants were told to increase the load once a week by 10% of the starting weight, or less if their pain intensified. We also gave them written instructions on how to perform the exercise. The patients were encouraged to do their exercise at home on a daily basis for the 12 weeks following enrollment. Further, a secretary at NIMI sent all included patients a weekly text message as a reminder to do their daily exercise.

#### Acupuncture

An acupuncturist with 12 years of clinical experience performed all the acupuncture treatments according to traditional Chinese methods [16]. For the acupuncture in this study, we gave a generalized treatment, consisting of selected local points recommended by an expert panel for the treatment of LE; we selected LI11 and LI10 over the muscular origin of the lateral extensor group of the forearm and LU5 in the cubical region. As distal points, we selected LI4 and TE5 for the treatment of pain in the upper limb, GB34 for treatment of tendinitis in general, and ST36 for treatment of pain [24]. The acupuncturist inserted the needles down to the musculature, approximately 15 mm in depth, to obtain a De Qi sensation. All the points except ST36 were manipulated with a reducing technique to obtain pain relief. The needles remained in situ for 20 min*.*

#### Manual therapy

Two physiotherapists with specific manual therapy qualifications and long clinical experience performed all the manual therapy sessions according to evidence-based physiotherapy. The manual therapy techniques consisted of Mulligan’s mobilization with movement (MWM) [25]. The manual therapists performed a lateral glide with gripping, a posterior-anterior glide on the radial head with supination of the radio-ulnar joint, and a lateral gapping manipulation technique. The mobilization techniques consisted of three sets of eight repetitions [25].

The eccentric exercise alone group was instructed once (on the day of randomization) and did not have any further contact with the therapists. They did receive a weekly text message by a secretary of NIMI, to be reminded to do their daily exercise at home. Patients in the acupuncture and manual therapy groups received their first treatment within 1 week of randomization. During a period of 12 weeks, they attended a minimum of three and a maximum of eight treatment sessions, depending on the patients’ perceived pain intensity and the therapists’ clinical evaluations. All groups received the same information and advice, including details about the natural course of the condition and expected duration of symptoms. Patients were encouraged to use their arm normally, but to avoid carrying heavy loads and pain-provoking activities such as gripping and repetitive wrist movement. Patients were allowed other kinds of treatments during the trial, except corticosteroid injections.

### Outcome measures

All clinical outcomes measures are standardized and validated patient-reported outcome measures (PROMs). Further, all outcome measures were retrieved at baseline before randomization. PROM data were captured electronically using Infopad, a web-based data capture system, compliant with all relevant regulations. The patients entered data in the Infopad application after receiving e-mails with links to the questionnaires. The research leader and those involved in the research project were blinded for the outcome results.

#### Primary outcomes

Retention was defined as the percentage of patients enrolled at baseline who completed all follow-up measures. Adherence was defined as patient’s commitment to treatment as recommended. Patient adherence to treatment was assessed using attendance at the treatment sessions. Documentation by the treating physical therapist or acupuncturist was used to quantify the total number of sessions completed.

#### Secondary outcomes

##### Numeric rating scale (NRS)

Secondary outcomes included patient-reported pain scores collected at weeks 1, 2, 3, 4, and 12 and 1 year after the start of treatment. The patients used an NRS to assess the intensity of their elbow pain. The NRS ranges from 0 to 10, with a lower score indicating less pain. All patients completed three scales at the given time points, to report on their present condition and their highest and lowest levels of pain during the last week; these answers were used to calculate an average score. The NRS assessment tool is found to be valid and a reliable method for measuring patients’ perceived pain [26].

##### Disabilities of the arm, shoulder, and hand (DASH)

The level of disability of the elbow was assessed with Quick-DASH, which is a shorter version of the original DASH, collected at weeks 4 and 12 and 1 year after the start of treatment.

Study results indicate that the Quick-DASH can be used instead of the original DASH with similar precision for upper extremity disorders [27, 28].

Patients were also asked to report days of sick leave and use of analgesics, and they reported their satisfaction with treatment and global perceived effect at the 12-week follow-up [29].

### Sample size calculation

As the data in this report were obtained to generate preliminary estimates of treatment effectiveness, no a priori sample size calculation was performed to ensure sufficient statistical power to detect between-group differences in treatment effect [30].

### Statistical methods and analysis

We analyzed all data using Statistical Program for Social Sciences (SPSS), version 22. Descriptive statistics were used to describe the primary outcomes of retention rates and treatment adherence [31]. Primary and secondary outcomes were analyzed group wise at given time points. Differences between groups were assessed both at given time points and when all measurements were considered. All available data were analyzed using linear mixed models for repeated measures, with an unstructured covariance matrix to model dependencies within individuals assessed at multiple time points. Mixed models allow for the assessment of possible differences between groups, adjusted for selected covariates and when all time points are considered [31]. In addition, the estimated differences between groups can be calculated for given time points. The model was adjusted for the possible confounders: age, gender, level of education, outcome, and time. The results are presented as the estimated overall means with 95% confidence intervals (CI). All statistical tests were two-sided. *p* values < 0.05 were considered statistically significant. As this is a pilot study, our results were considered exploratory and no correction for multiple testing was performed [31].

## Results

### Recruitment and participant flow

Fifty patients were referred to the study between April 2015 and May 2016. Of these, 14 were excluded (12 did not meet the inclusion criteria and 2 declined to participate). Therefore, 36 patients were included and randomized in the pilot study. In total, 13 patients were randomized to treatment with acupuncture, 12 to manual therapy, and 11 to eccentric exercise alone. All patients received the allocated treatment. Patients in the acupuncture and manual therapy groups concluded the treatment in accordance with protocol (attending at least three of a maximum of eight treatment sessions).

**Fig. 1:**
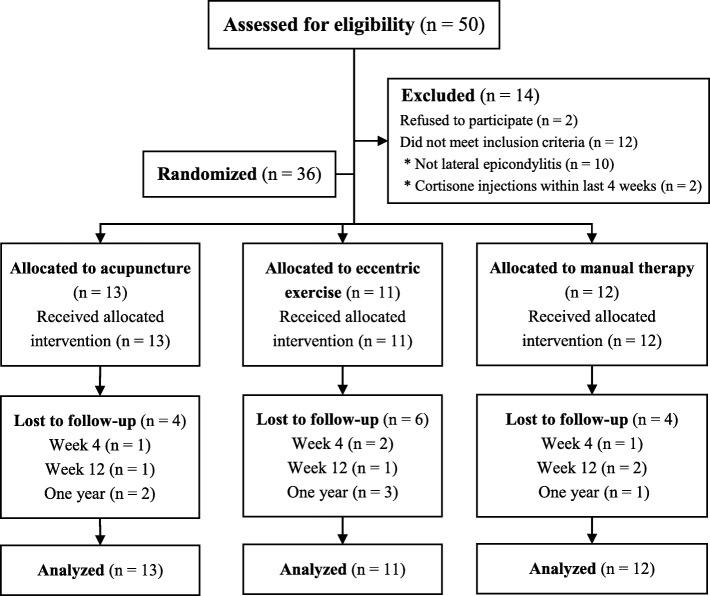
Recruitment and participant flow

The trial was completed in August 2017, with 22 (61%) patients completing all measurements including the final follow-up 1 year after the start of treatment. The majority of patients lost to follow-up were in the group of exercise alone. A few patients were unreachable, but the rest gave reasons as lack of time and unwillingness to answer the questionnaires. One patient in the manual therapy group reported problems with the questionnaires and, for that reason, wanted to withdraw from the trial. Another patient in the manual therapy group reported use of corticosteroid injection and withdrew from the trial at week 12. Figure 1 summarizes the patient flow.

### Baseline data

The groups were similar in terms of mean age, work status, and overall severity of symptoms (Table 1); however, a higher proportion of patients in the manual therapy group were male (75%), had higher education (92%), and were office workers (83%), compared to the other two groups. The patients in the acupuncture group had lower mean pain scores at baseline, compared to the other two groups.

**Table 1:** Characteristics of study population at baseline

Variable		Total (*N* = 36)	Exercise alone (*N* = 11)	Acupuncture (*N* = 13)	Manual therapy (*N* = 12)
Age (years)	Mean (SD)	49 (11)	47 (3)	51 (4)	49 (2)
Female		15 (42)	6 (55)	6 (46)	3 (25)
Higher education*		29 (81)	8 (73)	10 (76)	11 (92)
Type of work					
- Manual		12 (33)	5 (45)	5 (38)	2 (16)
- Office		24 (67)	6 (55)	8 (62)	10 (83)
Work status					
- Paid work		30 (82)	10 (90)	10 (77)	11 (92)
- Sick leave		2 (6)	–	1 (8)	1 (8)
- Student		2 (6)	–	–	–
- Retired		1 (3)	–	–	–
- Unemployed		1 (3)	–	–	–
Previous treatment					
- Cortisone injections		6 (17)	1 (9)	2 (15)	3 (25)
Pain intensity**	Mean (SD)	4.3 (1.3)	4.8 (1.3)	3.9 (1.3)	4.1 (1.3)
Functional capacity of arm***	Mean (SD)	30.5 (14.2)	29.2 (14.4)	30.5 (14.2)	31.7 (15.2)

Values are numbers (percentages) unless otherwise stated

*College or university degree (3 years or more)

**NRS (0–10). 0 = no pain, 10 = worst pain. Unadjusted mean

***Quick-DASH (0–100). A higher score indicates greater disability. Unadjusted mean

### Outcomes and estimations

#### Primary outcomes

Retention rates were similar for the treatment groups, but worse in exercise alone. Nine patients in the acupuncture group completed the 1-year follow-up, compared to eight patients in manual therapy group and five in exercise alone.

##### Number of treatment sessions

The mean number of treatment sessions attended (with 95% CI) was 3.92 (2.98; 4.86) for the acupuncture group and 4.33 (3.17; 5.49) for the manual therapy group. The majority of participants (64%) in both treatment groups received only three treatment sessions, which was the minimum number required in the trial (maximum of eight); the reasons included non-attendance or recovery from pain.

#### Secondary outcomes

##### Patient-reported pain

**Fig. 2:**
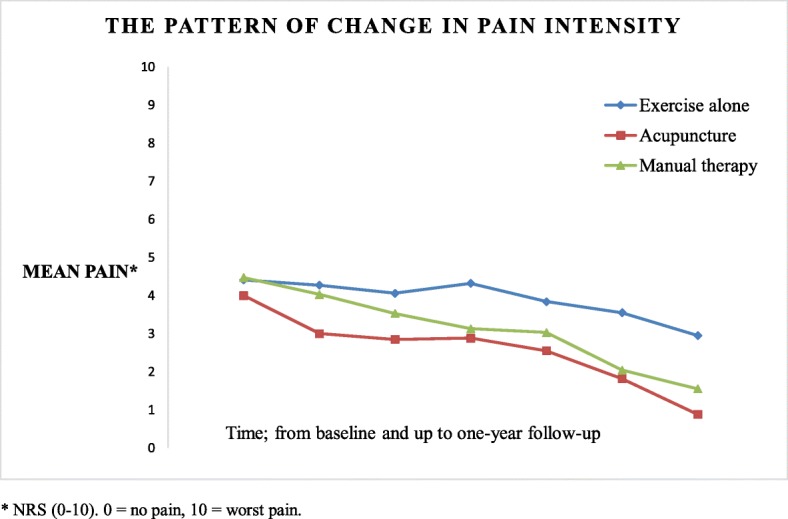
The pattern of change in pain intensity from baseline to last follow-up for all groups. Blue line, exercise alone; green line, manual therapy; red line, acupuncture. Data shown as mean pain using NRS (0–10): 0, no pain; 10, worst pain

Adjusted mean pain scores estimated with 95% CI at all follow-up time points are listed in Table 2. The acupuncture and manual therapy groups showed a gradual and very similar pattern of pain relief, while exercise alone showed smaller improvement. Patients in exercise alone had significantly higher mean pain scores than those in the acupuncture group. The estimated mean pain score (with 95% CI) was 4.42 (3.50; 5.36) for exercise alone (all measurements considered), 2.72 (1.97; 3.47) for acupuncture, and 3.25 (2.35; 4.16) for the manual therapy group. The pattern of change in pain intensity from baseline to last follow-up for all groups is depicted in Fig. 2.

**Table 2:** Adjusted mean scores of pain intensity and functional capacity of arm, estimated with 95% CI at each follow-up

Outcome measures	Follow-up	Exercise alone (*N* = 11)	Acupuncture (*N* = 13)	Manual therapy (*N* = 12)
Pain score*	Baseline	4.41 (3.66; 5.16)	4.00 (3.33; 4.67)	4.47 (3.73; 5.20)
	1 week	4.27 (3.33; 5.20)	3.00(2.20; 3.80)	4.03 (3.09; 4.97)
	2 weeks	4.06 (2.90; 5.21)	2.85 (1.90; 3.79)	3.53 (2.43; 4.63)
	3 weeks	4.32 (3.31; 5.33)	2.88 (2.07; 3.69)	3.13 (2.21; 4.05)
	4 weeks	3.84 (2.79; 4.89)	2.55 (1.65; 3.45)	3.03 (2.02; 4.04)
	12 weeks	3.55 (2.40; 4.68)	1.82 (0.92; 2.72)	2.04 (0.86; 3.20)
	1 year	2.95 (1.72; 4.18)	0.88 (0.03; 1.79)	1.55 (0.49; 2.60)
Function of arm**	Baseline	31.54 (22.10; 40.98)	33.72 (25.44; 41.99)	37.24 (27.89; 46.59)
	4 weeks	35.41 (25.35; 45.47)	28.69 (20.38; 37.00)	28.73 (18.92; 38.55)
	12 weeks	30.00 (19.96; 40.04)	19.81 (11.99; 27.63)	16.08 (05.56; 26.60)
	1 year	28.15 (16.18; 39.47)	12.19 (02.87; 21.52)	14.83 (04.22; 25.44)

*NRS (0–10). 0 = no pain, 10 = worst pain

**Quick-DASH (0–100). A higher score indicates greater disability

To compare the groups statistically, estimated marginal means for pain relief, adjusted for covariates, such as age, gender, level of education, outcome, and time, were analyzed with a linear mixed model. The result of the analyses was significant for differences in mean pain relief for the treatment groups compared to exercise alone, all measurements considered. Further, the acupuncture group was significantly (*p* < .001) and highly different from the exercise alone group. However, the difference in estimated mean pain relief between the acupuncture and manual therapy groups was only borderline significant (*p* < .040) (all measurement considered). The presentation of pairwise comparison of all groups is in Table 3.

**Table 3:** Mean difference between groups in pain intensity, all measurements considered (all estimated differences were statistically significant)

Mean difference between groups	Mean difference^*^	95% CI
At 1-year follow-up		
Difference between manual therapy and acupuncture	0.54	0.24;1.06
Difference between exercise alone and acupuncture	1.35	0.83;1.86
Difference between exercise alone and manual therapy	0.80	0.24;1.36

^*^NRS (0–10). 0 = no pain, 10 = worst pain

##### Level of disability of the elbow and arm

Adjusted mean scores of level of disability of the elbow and arm, estimated with 95% CI at all follow-up time points, are listed in Table 2. The acupuncture and manual therapy groups showed a gradual and very similar pattern of improvement in the function of the elbow and arm, while exercise alone showed lesser improvement. Patients in exercise alone had higher levels of disability of the elbow and arm than those in the treatment groups; the estimated mean score of level of disability of the elbow and arm (with 95% CI) was 31.66 (1.72; 41.60) for exercise alone (all measurements considered), 24.39 (16.34; 32.43) for acupuncture, and 26.97 (16.86; 37.08) for manual therapy. Overall, with all measurements considered, all participants improved their arm function; however, our data did not reveal any between-group differences.

##### Patient satisfaction

The patients were asked to report their satisfaction with treatment at 12-weeks follow-up. Twenty-five (69%) patients answered these questions, of which 21 (84%) were satisfied with the treatment. Only one patient in the group of exercise alone was not satisfied, and three were indifferent. Furthermore, they were asked to report how much their condition had improved or deteriorated since the start of treatment (global perceived effect). The majority of all patients reported that their condition had improved; two patients in the group of acupuncture reported a complete recovery, and one patient in the group of exercise alone reported that his condition had worsened.

## Discussion

To our knowledge, there have been no clinical trials investigating the clinical comparative effectiveness of acupuncture versus manual therapy in the treatment of LE. Feasibility studies play an important role in the planning of RCTs for novel interventions or a combination of existing interventions in new patient populations or recruitment settings. We demonstrated acceptable retention rates for patients in treatment groups; however, the retention of patients randomized to eccentric exercise alone was slightly worse. Patient adherence and satisfaction with treatment were good. Secondary outcomes suggest that treatments in addition to eccentric exercise may result in less pain and decreased disability for patients with LE. This study has confirmed the feasibility of executing a larger trial, with some changes to improve retention rates, to examine treatment effects more precisely.

Our goal was to demonstrate a retention rate above 80% to avoid serious threats to validity, but the result was lower than expected. Nine patients in the acupuncture group completed the 1-year follow-up, compared to eight in the manual therapy group and five in exercise alone. One factor that may have contributed to the low retention rate in the exercise alone group is that they were instructed only once (on the day of randomization) and did not have any further contact with the therapists. They did receive a weekly text message, from a secretary at NIMI, to be reminded to do their exercise. A possible explanation for the higher retention rates in the treatment groups, compared to exercise alone, could be that the patients had repeated contact with their manual therapist or acupuncturist. When receiving treatment in addition to home exercise and interaction with the therapist in a clinical setting, the motivation and likelihood of answering the questionnaires is higher. One way to improve the retention rates could be to maintain contact with the patients in the exercise alone group throughout the intervention. The patients could also be encouraged to share their concerns with a therapist or research leader via e-mail. Further, all patients could be encouraged to make contact between the end of treatment (at 12 weeks) and final follow-up (at 1 year), in an attempt to improve retention rates in all groups.

The patients reported their pain intensity and other symptoms electronically using PROMs. This method of data collection makes it possible to have several measurements and follow-ups. On the other hand, objective assessments such as a pain-free grip test measured with a dynamometer would have elucidated our findings and possibly improved retention rates for all groups. Hence, further research, as in a full-scale RCT, is required to determine if the treatments are effective when using objective measurements in addition to PROMs.

All patients received the allocated treatment. Further, patients in the acupuncture and manual therapy groups concluded the treatment in accordance with protocol (attending at least three of a maximum of eight treatment sessions), but the majority of patients (64%) in both treatment groups attended no more than the minimum number of sessions, which is less than expected. Patients were not compensated for participating in the study and, as under normal circumstances, had to pay for their own treatments, which may have contributed to lower attendance than expected. There may be need for a measure of affordability, as in income or insurance cover at baseline in a future fully scaled trial. On the other hand, a majority of the patients reported pain relief, or recovery from pain, after attending the minimum number of treatments required in the trial, which is a better outcome than expected.

To track adherence to treatments, we asked the patients to report their satisfaction with treatment sessions at 12-week follow-up. Twenty-five (69%) patients answered these questions, of which 21 (84%) were satisfied with the treatment. This result is better than expected and could be a positive reflection of the therapist-patient relationship, treatment effectiveness, and/or the clinical setting of this trial. The project was ambitious, with several treatment modalities in a clinical setting, making it impossible to control for all confounding variables. With access to both patients and therapists in a clinical setting at NIMI, it was more natural and practical to explore the effectiveness, rather than the efficacy, of LE treatments [32, 33]. Effectiveness refers to a pragmatic trial, seeking answers to whether an intervention will work under normal conditions [34].

Our aim was to assess feasibility; therefore, we did not expect to find statistical differences in our secondary outcome measures. Nevertheless, some treatment effect sizes between groups were greater than anticipated, especially between acupuncture and exercise alone. This could indicate a possible clinical effect of acupuncture and manual therapy treatment in addition to eccentric exercise for LE. The pattern of effect seems to follow the theory that acupuncture and manual therapy could enhance the effect of exercise and/or speed the healing of the affected tendon [9, 10]. Both acupuncture and manual therapy induce analgesia through several pain mechanisms, which allow for exercise and load management to increase strength [15, 35]. In addition to pain relief, acupuncture has also shown the potential to increase local blood flow within a target tissue and affect fibroblast migration through myofascial collagen stimulation, both important aspects of the healing process of the affected tendon [36].

During acupuncture or manual therapy, patients benefit not only from the treatment itself, the needling, or the manipulation techniques, but also from the non-treatment specific factors [36].

Important non-treatment-specific factors can be expectancy, motivation, and other psychosocial aspects such as therapist-patient relationships [36]. It is important to consider these factors when discussing our secondary outcomes. Thus, although those in treatment groups reported greater pain relief than the eccentric exercise alone group, some of their pain relief could be explained by the non-treatment-specific effect. The effect of the positive expectations of patients in treatment groups could improve their outcomes. On the other hand, negative expectations of patients in exercise alone, because they are not receiving additional treatment, could cause the treatment to have a more negative effect than it would otherwise have had, creating a nocebo effect. When receiving additional treatment and interaction with a therapist in a clinical setting, the motivation and likelihood of completing the exercise program at home is higher. The outcomes of strength exercise programs seem to depend on the patients’ motivation and compliance [37].

Based on previous publications, we expect the minimal important change (MIC) in pain from baseline to the end of the trial to be a reduction of 2 points on the NRS [38]. The result of the explorative statistical analysis was that patients receiving treatment, either acupuncture or manual therapy, in addition to eccentric exercise, experienced greater pain relief than those receiving eccentric exercise alone. The difference between acupuncture and exercise alone was highly statistically significant (*p* < .000), with a mean change in pain score of 1.35 (0.26). However, to our knowledge, there is no clear definition of the size of between-group differences, and when they should be viewed as clinically significant—meaning that one treatment is clinically better than the other(s) [39]. The explorative analysis also revealed some differences in pain relief between acupuncture and manual therapy, but these differences were very small and of uncertain clinical value. Narrow CI may explain statistically significant differences between groups despite clinical changes from baseline to end of study just under the pre-specified magnitude, and differences in pain and disability scores between treatment groups of uncertain clinical significance.

### Risks

No participants reported adverse advents or harm. Both acupuncture and manual therapy are considered safe treatments and are among the most common physical interventions for pain relief.

### Limitations and strengths

Our study was designed to assess the feasibility of implementing manual therapy and acupuncture, in addition to eccentric exercise, for patients with LE; therefore, we cannot make any definitive statements regarding the effectiveness of these treatments. A strength of this study is the fact that the participants were recruited from among tennis elbow patients in an outpatient health care center specializing in sports medicine and rehabilitation of musculoskeletal conditions. Although the participants do not represent a random sample, they may be regarded as representative of this type of patient in the general population. We included patients with symptom durations of 2 weeks or longer. If we had distinguished between the acute and chronic stages of tendinopathy, we could have adjusted for the difference in duration of symptoms in the statistical analysis, because duration of symptoms, related to the different stages of tendinopathy, might affect the outcome [8]. The comorbidities of neck and shoulder pain were selected as exclusion criteria, since the associated patients are reported to have a poorer prognosis in regard to duration of symptoms of LE and treatment outcomes [3, 8]. An inherent limitation of this study is that we did not log or supervise the performance of exercise. Therefore, we have no certain information on whether the patients performed their daily exercise or not. In an optimal clinical and trial setting, we would prefer to give the patients an exercise diary and supervise some of the exercise performance, in order to allow for optimal management of load progression and to balance the effect of therapy between the groups [37]. Based upon experience with strength exercise for tendinopathy at NIMI, the load progression should be slow and performed below the patient’s pain threshold. To our knowledge, there is no research exploring the role that supervision of exercise plays in terms of patient compliance.

### Clinical implications and further research

There is a need to clarify the role of exercise in the management of LE, including optimal type and dosage of exercises. Although exercise is the cornerstone of rehabilitation, it has received less research attention than other interventions in the treatment of LE. There is a need for future RCTs investigating the effect of exercise for patients with LE, especially in combination with other physical modalities, and the role of supervision of exercise in terms of patient compliance.

## Conclusions

These results are based on a small study population (feasibility trial), with much less power than calculated for a full-scale trial, and with clinical changes from baseline to end of study just under the pre-specified magnitude, and differences in pain and disability scores between treatment groups of uncertain clinical significance. Therefore, we must treat these findings with caution. The overall effect of treatment is, however, statistically significant and almost clinically significant; therefore, it would be worthwhile to follow up the present feasibility trial with a larger trial. Based on our secondary outcomes and retention and adherence rates, a full-scale RCT appears feasible and warranted to assess treatment effects more precisely.

## Data Availability

De-identified individual-patient data are available by contacting the corresponding author.
